# Supplementary material for: EV-Associated miRNAs from Peritoneal Lavage are a Source of Biomarkers in Endometrial Cancer
Source: Cancers (Basel). 2019 Jun 18;11(6):839. doi: 10.3390/cancers11060839 (PMC6628347; doi:10.3390/cancers11060839)
Supplement: Supplementary file 1 [file cancers-11-00839-s001.zip › cancers-505924-suppl-final/cancers-505924-figure s1.docx]

Supplementary Materials: EV-Associated miRNAs from Peritoneal Lavage is a Source of Biomarkers in Endometrial Cancer

Berta Roman-Canal, Cristian Pablo Moiola, Sònia Gatius, Sarah Bonnin, Maria Ruiz-Miró, Esperanza González, Xavier González-Tallada, Ivanna Llordella, Isabel Hernández, José M. Porcel, Antonio Gil-Moreno, Juan M. Falcón-Pérez, Julia Ponomarenko, Xavier Matias-Guiu and Eva Colas


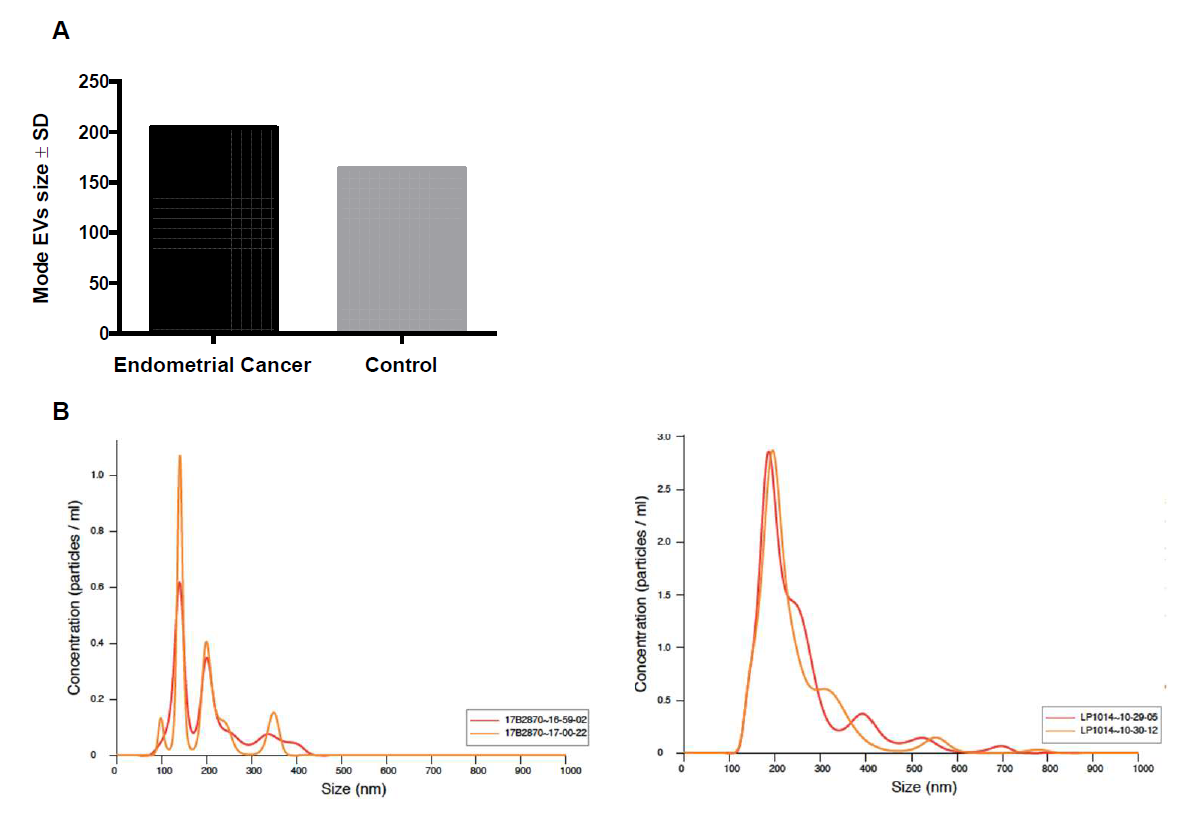


**Figure S1.** EVs characterization. (**A**) Box-plot representing the average mode of EVs isolated from the peritoneal lavage and ascitic fluid of EC and control patients, respectively (Mean ± SD); measured by Nanoparticle Tracking Analysis. (**B**) Size distribution and concentration of isolated EVs of a peritoneal lavage of a EC patient (left) and a ascitic fluid of a control patient (right), measured by Nanoparticle Tracking Analysis.
